# YELLOW ALERT: Persistent Yellow Fever Virus Circulation among Non-Human Primates in Urban Areas of Minas Gerais State, Brazil (2021–2023)

**DOI:** 10.3390/v16010031

**Published:** 2023-12-23

**Authors:** Gabriela F. Garcia-Oliveira, Anna Catarina Dias Soares Guimarães, Gabriel Dias Moreira, Thais Alkifeles Costa, Matheus Soares Arruda, Érica Munhoz de Mello, Marlise Costa Silva, Munique Guimarães de Almeida, Kathryn A. Hanley, Nikos Vasilakis, Betânia Paiva Drumond

**Affiliations:** 1 Laboratório de Vírus, Departament of Microbiology, Universidade Federal de Minas Gerais, Belo Horizonte CEP 31270-901, Brazil; gabrielafernandag@gmail.com (G.F.G.-O.); annacatarinaanna@gmail.com (A.C.D.S.G.); gabriel.dias05082000@gmail.com (G.D.M.); alkifelesthais@gmail.com (T.A.C.); matheusmtsa095@gmail.com (M.S.A.); 2 Centro de Controle de Zoonoses, Prefeitura de Belo Horizonte, Belo Horizonte CEP 31270-705, Minas Gerais, Brazil; 3 Laboratório de Zoonoses, Prefeitura de Belo Horizonte, Belo Horizonte CEP 31270-705, Minas Gerais, Brazil; 4 Department of Biology, New Mexico State University, Las Cruces, NM 88003-8801, USA; khanley@nmsu.edu; 5 Department of Pathology, University of Texas Medical Branch, Galveston, TX 77555-0609, USA; nivasila@utmb.edu; 6 Center for Vector-Borne and Zoonotic Diseases, The University of Texas Medical Branch, Galveston, TX 77555-0609, USA; 7 Institute for Human Infection and Immunity, University of Texas Medical Branch, Galveston, TX 77555-0610, USA

**Keywords:** Brazil, yellow fever, yellow fever virus, epizootics, outbreaks, non-human primate, surveillance, *Orthoflavivirus*

## Abstract

Yellow fever virus (YFV) is the agent of yellow fever (YF), which affects both humans and non-human primates (NHP). Neotropical NHP are highly susceptible to YFV and considered sentinels for YFV circulation. Brazil faced a significant YF outbreak in 2017–2018, with over 2000 human cases and 2000 epizootics cases, mainly in the State of Minas Gerais, Brazil. This study aimed to investigate whether YFV circulation persisted in NHP after the human outbreak had subsided. To this end, NHP carcass samples collected in Minas Gerais from 2021 to 2023 were screened for YFV. RNA was extracted from tissue fragments and used in RT-qPCR targeting the YFV 5’UTR. Liver and lung samples from 166 animals were tested, and the detection of the β-actin mRNA was used to ensure adequacy of RNA isolation. YFV RNA was detected in the liver of 18 NHP carcasses collected mainly from urban areas in 2021 and 2022. YFV positive NHP were mostly represented by *Callithrix,* from 5 out of the 12 grouped municipalities (mesoregions) in Minas Gerais state. These findings reveal the continued YFV circulation in NHP in urban areas of Minas Gerais during 2021 and 2022, with the attendant risk of re-establishing the urban YFV cycle.

## 1. Introduction

Mosquito-borne yellow fever virus (YFV) (*Orthoflavivirus flavi*) (family *Flaviviridae*, *genus Orthoflavivirus*) is the etiologic agent of yellow fever (YF), an acute viral hemorrhagic and viscerotropic disease in both human and non-human primates (NHPs). YFV is currently endemic in tropical regions of Africa and South America. There are three known transmission cycles of YFV; in the sylvatic (jungle) cycle, NHPs serve as the primary reservoirs and amplification hosts, while humans are incidental hosts and mosquitoes of the genera *Aedes* (in Africa), *Sabethes*, and *Haemagogus* (in the Americas) serve as vectors. In the urban transmission cycle, humans are the primary hosts and the anthropophilic mosquito *Aedes aegypti* is the vector. Finally, the rural transmission cycle has been initially described in Africa and occurs at the interface between urban and rural or forested land covers. *Aedes* mosquitoes are implicated in the rural cycle, giving rise to isolated rural epidemics, which have the potential to trigger broader urban outbreaks [1,2,3,4,5].

**Figure 1:**
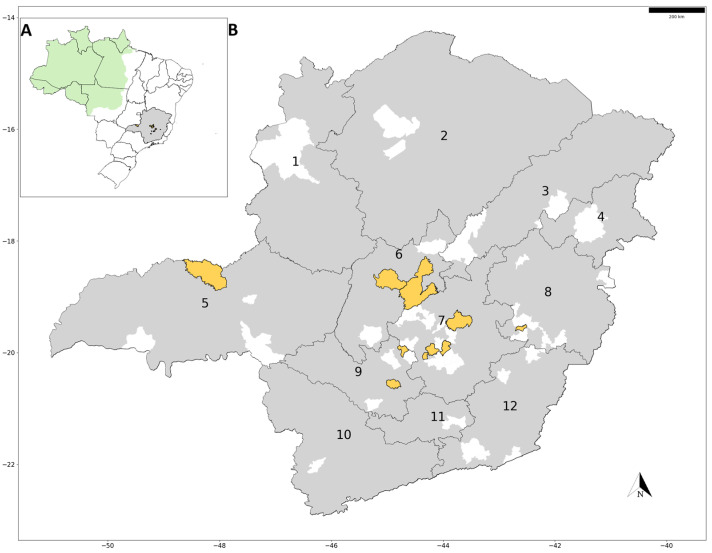
Detection of yellow fever virus RNA in carcasses of non-human primates in Minas Gerais state, Brazil, 2021–2022. (**A**) Map of Brazil outlines the states (black lines) and highlights the Amazon basin (in green) and Minas Gerais state (in grey). (**B**) In detail, the map of Minas Gerais state displays mesoregion boundaries (black lines), municipalities where YFV RNA was detected in NHP in this study (yellow), and municipalities where YFV was not detected in NHP in this study (white). The 12 mesoregions of Minas Gerais state are shown as follows: 1: Northwest; 2: North; 3: Jequitinhonha; 4: Mucuri Valley; 5: Triangulo Mineiro; 6: Central; 7: Metropolitan; 8: Rio Doce Valley; 9: Western; 10: South/Southwest; 11: Campo das Vertentes; 12: Zona da Mata. The map was generated using the geobr package, IBGE shapefile for Brazilian hydrographic basins (https://www.ibge.gov.br/geociencias/downloads-geociencias.html?caminho=informacoes_ambientais/estudos_ambientais/bacias_e_divisoes_hidrograficas_do_brasil/2021/Divisao_Hidrografica_Nacional_DHN250/vetores/ accessed on 13 October 2023), and Python 3.11.5.

Between the end of the urban YFV cycle in Brazil in 1942 up to 2000, the YFV sylvatic cycle was largely confined to the Amazon basin (Figure 1), although the virus sporadically re-emerged outside this region, especially during rainy periods accompanied by elevated temperatures [5,6]. Several factors underpin the reemergence of YFV in Southern Brazil, including inadequate vaccination coverage; the abundance of sylvatic vectors and NHP hosts; favorable climatic conditions; the emergence of new virus lineages; and the movement of infected humans and NHPs into new areas sharing these conducive conditions [6]. In addition, during the recent YF outbreaks in Brazil, the observed temporal and geographical patterns of YFV dispersion indicated the important role of vectors in YFV movement [7,8]. From late 2016 to 2018, Brazil experienced a dramatic shift in YF epidemiology, with a major outbreak in the Southeast region of the country. The state of Minas Gerais (MG) (Figure 1) was recognized as the epicenter of the epidemic. From July 2016 to June 2018, the outbreak resulted in more than 2000 human cases, with 760 deaths in the country. More than 2000 confirmed infections in NHP were documented during this time, including NHP carcasses sampled in urban areas, and current evidence suggests that all human cases were related to the sylvatic cycle [5,9,10,11,12,13].

After the outbreak and extending into the current monitoring period (from July 2023 to June 2024), vectors, NHPs, and human cases of YFV infection have been continuously reported outside the Amazon Basin in Brazil [14,15,16,17,18,19,20,21]. These reports underscore the ongoing circulation of YFV in Southern Brazil. Thus, we undertook an investigation of YFV infection within NHP carcasses collected from February of 2021 to August of 2023 from different covers, including urban settings, in MG. 

## 2. Materials and Methods

### 2.1. Study Area and Samples

MG state is located in Southeast Brazil, bordering the states of São Paulo, Rio de Janeiro, and Espírito Santo (Southeast Brazil), Bahia (Northeast Brazil), and Mato Grosso do Sul and Goiás (Midwest Brazil) (Figure 1A). It has a total population of approximately 20.5 million inhabitants concentrated (85%) in urban areas that correspond to 0.8% of its total territory (586,513,983 km^2^) [22]. MG state is subdivided into 12 mesoregions based on the economic and social traits of the municipalities (Figure 1B). The state is covered by three main biomes: Atlantic Forest, Cerrado (Brazilian savannah), and Caatinga. Different genera of NHPs have been described in the state as *Alouatta*, *Callithrix*, *Sapajus*, *Callicebus*, and *Brachyteles* [23,24]. 

The molecular investigation of YFV was carried out using liver and lung samples obtained from 166 carcasses of free-living NHPs collected in 54 municipalities across MG state from February 2021 to August 2023 (Table 1). The carcasses were collected by health surveillance agents as part of the YF surveillance program and forwarded to the Laboratory of Zoonosis of Belo Horizonte, MG, where all carcasses were tested as negative for rabies. The collection of NHP carcasses spanned both the rainy (October to March) and dry (April to September) seasons. This collection encompassed urban, urban–rural transition, and rural/forested areas in 11 mesoregions within MG state (Figure 1B, Table 1). Information was obtained from epidemiological records regarding the address or geographic coordinates where carcasses were collected and used to classify the carcasses based on land use as urban (built-up) areas, peri-urban areas, and rural/forested areas, as previously described [11]. Among the 166 carcasses studied, a majority (89.2%) were identified as *Callithrix* genus, while a smaller portion belonged to *Alouatta* genus (2.4%) or the Cebidae family (3.6%), while some carcasses (4.8%, *n* = 8) could not be definitively identified (Table 1).

### 2.2. YFV Screening

Fragments of 20 to 30 mg of tissues (lung and liver), previously obtained from the NHP carcasses and preserved in RNAlater™ Stabilization Solution (Invitrogen—Thermo Fisher Scientific, Waltham, MA, USA) at −80 °C, were separately used for total RNA extraction (RNeasy^®^ Mini Kit total RNA extraction kit—QIAGEN, Venlo, Holanda). The fragments were placed in screw-cap tubes containing 600 µL of the lysis solution, provided by the kit, and three 2 mm borosilicate beads (Sigma-Aldrich, San Luis, MO, USA). They were then agitated for two minutes using a bead beater (Mini-Beadbeater-16, BioSpec Products, Bartlesville, OK, USA) to ensure homogeneity for the next steps. RNA extraction was performed in batches of up to 13 samples plus a negative extraction control (nuclease-free water). 

One-step RT-qPCR (GoTaq^®^ Probe 1-step RT-qPCR System kit, Promega Corporation, Madison, WI, USA) for endogenous control β-actin coding gene (Table S1) [25] was performed to confirm the viability of total RNA extracted. All samples were positive, showing suitability to be used as templates for RT-qPCR. Each RNA sample, along with the negative extraction controls (2.5 µL), were screened in duplicate for YFV RNA by RT-qPCR (GoTaq^®^ Probe 1-Step RT-qPCR System kit, Promega Corporation, Madison, WI, USA) with primers and probe (Table S1) targeting the 5’ untranslated region of YFV viral genome [26]. In addition, a non-template control (nuclease-free water) and a positive control (YFV 17DD RNA, kindly provided by Dr. Pedro Augusto Alves from FIOCRUZ Minas Gerais) were used in each set of RT-qPCR. Samples that presented a cycle threshold for amplification (Cq) ≤ 37 in duplicate or >40 were confidently considered positive and negative for YFV, respectively. Samples with Cq values > 37 and ≤40 were initially categorized as indeterminate and subjected to retesting. They were only classified as positive if they yielded consistent positive results in duplicate with Cq values ≤ 40 during the second round of testing.

To ensure accuracy, all samples that initially tested positive for YFV RNA underwent further testing. This involved employing the same primers and probes for YFV in two distinct conditions: RT-qPCR (with reverse transcriptase) and PCR (without reverse transcriptase). This comprehensive approach was implemented to eliminate the possibility of amplicon carryover. In addition, aiming to genotype [25] YFV, RNA from the positive samples were used as templates in RT-qPCR using a pan-orthoflavirus protocol targeting the NS5 genome region [27] (Table S1).

## 3. Results and Discussion

Liver and lung samples from 166 NHP carcasses collected over a span of three years (February 2021 to August 2023) were tested for the presence of YFV RNA. Liver samples from 18 NHP carcasses were positive (Table S2) while all lung samples were negative. Positive samples showed Cqs ranging from 33.4 to 37.3 with an average Cq of 36.1. The 18 positive samples only showed amplicons when used in RT-qPCR, with no amplicons being detected in PCR reactions (in absence of reverse transcriptase). Positive RNA samples were obtained from five different RNA extraction batches, each one with a negative extraction control. All non-template controls and negative RNA extraction controls showed negative results for RT-qPCR. Unfortunately, none of the positive samples yielded amplicons suitable for further genotyping. The inability to generate NS5 fragment amplicons for genotyping may be related to either RNA degradation or the low viral load of the samples indicated by the high Cq values. 

The greater detection of YFV in NHP carcasses collected during the dry season may be attributable to a sampling bias. This bias stems from the fact that the carcasses were collected as part of the passive surveillance of YF. In addition, the lower humidity and cooler temperatures observed during the dry season could contribute to reduced carcass decomposition rates and, subsequently, an increased likelihood of sampling [28]. The majority of the 18 positive NHPs were *Callithrix penicillata* (89%), which were mostly obtained in urban areas (72%) (Table 1). The low YFV genomic loads observed in *Callithrix*, indicated by high Cq values in RTqPCR, were also previously described in other studies during periods of intense YFV circulation during the outbreaks [11,12,13], or during periods with low viral circulation after the outbreaks [18,20]. The lower viral loads observed in *Callithrix* compared to specimens of other genera could be related to their susceptibility to YFV and their role in YFV maintenance and transmission cycles should be further investigated [11,12,13]. The greater detection of YFV in *Callithrix* specimens may reflect the remarkable behavioral plasticity of this genus, enabling them to thrive in densely populated areas and urban environments [29], where some other species cannot persist. In fact, in a previous study, the majority of NHP carcasses (436 out of 452), sampled in urban areas during the epizootics of YF in 2017/2018 in MG, corresponded to *Callithrix* specimens, with an infection rate for YFV of 27% in several urban areas of MG [11]. Although we only detected YFV in NHP carcasses collected in 2021 *(n* = 10) and in 2022 (*n* = 8), data from the Health Department of MG confirmed a case of YF in a human being in 2023 [30]. The YFV-positive NHP carcasses originated from 6 out of 12 mesoregions of MG (Figure 1B, Table 1). Although vaccination coverage has increased within the human population since the last outbreaks, a concerning observation is that some of the mesoregions of Minas Gerais with YFV circulation have municipalities with YF vaccination coverage below the recommended threshold of 95% [17,31,32].

The regions where YFV was detected here were heavily affected during the YF outbreaks in 2017/2018, with subsequent records of YF in NHPs [31,32]. The enduring presence of YFV could be linked to the ongoing circulation of the virus associated with previous outbreaks [18] or to a new introduction of YFV in the region, as recently described in 2021 [20]. Silva and colleagues [18] have demonstrated the maintenance of the YFV lineage related to 2017/2018 outbreaks in the enzootic cycle in MG state up to 2020. Previous studies lend further support to the continued enzootic circulation of YFV within MG since the 2017/2018 outbreaks, in NHPs [18,20,31,32] and vectors [21] during rainy or dry seasons, and in rural/forested or urban areas, reinforcing the fact that YFV has suitable ecological conditions for its maintenance in the state [33]. These facts raise concerns related to the establishment of YFV enzootic cycles, including urban areas in Southeast Brazil, currently considered a non-endemic region for YF, with risks of viral spillover to the human population. Notably, during 2023, human cases confirmed by laboratory criteria, often involving individuals with a history of exposure to wild areas, have been reported in Minas Gerais, São Paulo (Southeast region), Paraná, and Rio Grande do Sul states (South region) [16,30]. 

There is an urgent need for further research focusing on YFV vectors, hosts, and their interactions in urban settings in Southern Brazil. Such studies are essential for gaining a deeper understanding of the dynamics of the YFV sylvatic cycle, particularly in non-endemic areas like MG and especially within urban environments. Our findings emphasize the critical importance of sustained surveillance and further studies of YFV circulation in urban areas in Southern Brazil to safeguard public health.

## Figures and Tables

**Table 1:** Non-human primate carcasses sampled and tested for yellow fever virus, Minas Gerais state, 2021–2023.

NHP		Year						Total	
		2021		2022		2023			
		Total	+	Total	+	Total	+	Total	+
Season	Rainy	13	6	18	1	11	0	42	7
	Dry	41	4	46	5	19	0	106	9
	NA *	3	0	5	2	10	0	18	2
Sample collection area	Urban	40	8	44	5	0	0	84	13
	Peri-urban	4	0	6	0	0	0	10	0
	Rural/forested	9	2	10	1	0	0	19	3
	NA *	4	0	9	2	40	0	53	2
Taxon	*Callithrix* spp.	52	10	59	6	37	0	148	16
	*Alouatta* spp.	1	0	2	0	1	0	4	0
	Cebidae	1	0	3	0	2	0	6	0
	NA *	3	0	5	2	0	0	8	2
State Mesoregion	Central	7	1	8	1	0	0	15	2
	Triangulo Mineiro	3	1	4	0	2	0	9	1
	Rio Doce Valley	3	1	10	0	8	0	21	1
	Campo das Vertentes	0	0	0	0	1	0	1	0
	Jequitinhonha	0	0	1	0	2	0	3	0
	Metropolitan	32	5	30	5	24	0	86	10
	North	1	0	3	0	0	0	4	0
	Northwest	1	0	1	0	0	0	2	0
	South/Southwest	0	0	1	0	1	0	2	0
	West	4	2	3	0	1	0	8	2
	Zona Da Mata	3	0	3	0	1	0	7	0
	NA *	3	0	5	2	0	0	80	2
Total		57	10	69	8	40	0	166	18

NA *: not available, missing information in the epidemiological investigation files. NHP: non-human primate, +: positive.

## Data Availability

All research data is shared within the manuscript and Supplementary Tables S1 and S2.

## Supplementary Materials

The following supporting information can be downloaded at: https://www.mdpi.com/article/10.3390/v16010031/s1; Table S1: Primers and probe for RT-qPCR. Table S2: Non-human primate carcasses obtained from 2021 to 2023, Minas Gerais, Brazil, and tested for the presence of yellow fever virus RNA.
