# Correction: Garcia-Oliveira et al. YELLOW ALERT: Persistent Yellow Fever Virus Circulation among Non-Human Primates in Urban Areas of Minas Gerais State, Brazil (2021–2023). *Viruses* 2024, *16*, 31

**DOI:** 10.3390/v16101527

**Published:** 2024-09-27

**Authors:** Gabriela F. Garcia-Oliveira, Anna Catarina Dias Soares Guimarães, Gabriel Dias Moreira, Thais Alkifeles Costa, Matheus Soares Arruda, Érica Munhoz de Mello, Marlise Costa Silva, Munique Guimarães de Almeida, Kathryn A. Hanley, Nikos Vasilakis, Betânia Paiva Drumond

**Affiliations:** 1 Laboratório de Vírus, Departament of Microbiology, Universidade Federal de Minas Gerais, Belo Horizonte CEP 31270-901, Brazil; gabrielafernandag@gmail.com (G.F.G.-O.); annacatarinaanna@gmail.com (A.C.D.S.G.); gabriel.dias05082000@gmail.com (G.D.M.); alkifelesthais@gmail.com (T.A.C.); matheusmtsa095@gmail.com (M.S.A.); 2 Centro de Controle de Zoonoses, Prefeitura de Belo Horizonte, Belo Horizonte CEP 31270-705, Minas Gerais, Brazil; 3 Laboratório de Zoonoses, Prefeitura de Belo Horizonte, Belo Horizonte CEP 31270-705, Minas Gerais, Brazil; 4 Department of Biology, New Mexico State University, Las Cruces, NM 88003-8801, USA; khanley@nmsu.edu; 5 Department of Pathology, University of Texas Medical Branch, Galveston, TX 77555-0609, USA; nivasila@utmb.edu; 6 Center for Vector-Borne and Zoonotic Diseases, The University of Texas Medical Branch, Galveston, TX 77555-0609, USA; 7 Institute for Human Infection and Immunity, University of Texas Medical Branch, Galveston, TX 77555-0610, USA

## Text Correction

There was an error in the original publication [1]. The original sentence was pertaining to urban NHP only, but after language revision, it gave the idea that we were talking about all NHPs studied by Sacchetto et al., 2020 (https://doi.org/10.1371/journal.pntd.0008658) (urban, rural, and periurban areas). Additionally, Reference 13 was cited instead Reference 11. A correction has been made to Results and Discussion, Paragraph 2:

“In fact, in a previous study, the majority of NHP carcasses (436 out of 452), sampled in urban areas during the epizootics of YF in 2017/2018 in MG, corresponded to *Callithrix* specimens, with an infection rate for YFV of 27% in several urban areas of MG [11].”

## Modification Acknowledgements

In the original publication, our partner Secretaria Municipal de Saúde de Belo Horizonte was not included in Acknowledgments. A correction has been made: 

“**Acknowledgments:** We would like to thank Secretaria Municipal de Saúde de Belo Horizonte, Laboratório de Zoonoses, and Centro de Controle de Zoonoses da Prefeitura de Belo Horizonte, Secretaria de Estado de Saúde de Minas Gerais, colleagues from Laboratório de Vírus/UFMG, Pró-Reitorias de Graduação, Pós-graduação, and Pesquisa from Universidade Federal de Minas Gerais/Brazil.”

The authors state that the scientific conclusions are unaffected. This correction was approved by the Academic Editor. The original publication has also been updated.
