# Supplementary material for: YELLOW ALERT: Persistent Yellow Fever Virus Circulation among Non-Human Primates in Urban Areas of Minas Gerais State, Brazil (2021–2023)
Source: Viruses. 2023 Dec 23;16(1):31. doi: 10.3390/v16010031 (PMC10818614; doi:10.3390/v16010031)
Supplement: Supplementary file 1 [file viruses-16-00031-s001.zip › viruses-2776348-supplementary.pdf]

Table S1: Primers and probe used in RT-qPCR

Region	Primer or probe	Sequence	Reference
β-actin	Forward primer	5' CCAACCGCGAGAAGATGA 3'	Rezende et al, 2019
	Reverse primer	5' CCAGAGGCGTACAGGGATAG 3'	
NS5	All_S primer	5' TACAACATGATGGGGAARAGAGARAA 3'	Patel et al, 2013
	All_S2 primer	5' GTGTCCCA GCCNGCKGTGTCATCWGC 3'	
5'UTR	YFallF primer	5' GCTAATTGAGGTGYATTGGTCTGC 3'	Domingo et al, 2012
	YFallR primer	5' CTGCTAATCGCTCAAMGAACG 3'	
	YFallP probe	5’ FAM-ATCGAGTTGCTAGGCAATAAACAC-TMR 3’	

Rezende, I.M.; Alves, P.A.; Arruda, M.S.; Gonçalves, A.P.; Oliveira, G.F.G.; Pereira, L.S.; Dutra, M.R.T.; Campi-Azevedo, A.C.; Valim, V.; Tourinho, R.; Oliveira, J.G.; Calzavara, C.E.; Said, R.F.D.C.; Kroon, E.G.; Martins-Filho, O.A.; Teixeira-Carvalho, A.; Drumond, B.P. Yellow Fever Virus Genotyping Tool and Investigation of Suspected Adverse Events Following Yellow Fever Vaccination. *Vaccines* **2019**, *7*, 1–10, doi:10.3390/vaccines7040206.

Domingo, C.; Patel, P.; Yillah, J.; Weidmann, M.; Méndez, J.A.; Nakouné, E.R.; Niedrig, M. Advanced Yellow Fever Virus Genome Detection in Point-of-Care Facilities and Reference Laboratories. *J. Clin. Microbiol.* **2012**, *50*, 4054–4060, doi:10.1128/JCM.01799-12.

Patel, P.; Landt, O.; Kaiser, M.; Faye, O.; Koppe, T.; Lass, U.; Sall, A.A.; Niedrig, M. Development of One-Step Quantitative Reverse Transcription PCR for the Rapid Detection of Flaviviruses. *Virol. J.* **2013**, *10*, 58, doi:10.1186/1743-422X-10-58.

Table S2: Non-human primate carcasses obtained from 2021 to 2023, Minas Gerais, Brazil, and tested for the presence of yellow fever virus RNA

ID	Date	Season	Year	RTqPCR	Taxon	Area	Municipality	State mesoregion
1118	01-Feb-21	rainy	2021	neg	*Callithrix penicillata*	Urban	Itaúna	West
1119	03-Feb-21	rainy	2021	neg	*Callithrix penicillata*	Urban	Nova Lima	Metropolitan
1128	12-Feb-21	rainy	2021	pos	*Callithrix penicillata*	Urban	Timóteo	Rio Doce Valley
1140A	18-Feb-21	rainy	2021	pos	*Callithrix penicillata*	Urban	Igaratinga	West
1138	04-Mar-21	rainy	2021	pos	*Callithrix penicillata*	Urban	Jaboticatubas	Metropolitan
1136	16-Mar-21	rainy	2021	pos	*Callithrix penicillata*	Urban	Belo Horizonte	Metropolitan
1134	18-Mar-21	rainy	2021	pos	*Callithrix penicillata*	Rural	Carmo Da Mata	West
1122	30-Mar-21	rainy	2021	pos	*Callithrix penicillata*	Urban	Belo Horizonte	Metropolitan
1132	23-Apr-21	dry	2021	pos	*Callithrix penicillata*	Urban	Araguari	Triangulo Mineiro
1124	26-Apr-21	dry	2021	pos	*Callithrix penicillata*	Rural	Curvelo	Central
1130	10-May-21	dry	2021	pos	*Callithrix penicillata*	Urban	Belo Horizonte	Metropolitan
1126	18-May-21	dry	2021	neg	*Callithrix penicillata*	Urban	Belo Horizonte	Metropolitan
1141	26-May-21	dry	2021	neg	*Callithrix penicillata*	Urban	Belo Horizonte	Metropolitan
1143	30-May-21	dry	2021	neg	*Callithrix penicillata*	Urban	Belo Horizonte	Metropolitan
1145	30-May-21	dry	2021	neg	*Callithrix penicillata*	Urban	Belo Horizonte	Metropolitan
1155	01-Jun-21	dry	2021	neg	*Callithrix penicillata*	Urban	Itapajipe	Triangulo Mineiro
1165	01-Jun-21	dry	2021	neg	*Alouatta spp.*	Urban	Além Paraíba	Zona da Mata
1169	08-Jun-21	dry	2021	neg	*Callithrix penicillata*	Rural	Curvelo	Central
1171	08-Jun-21	dry	2021	neg	*Callithrix penicillata*	Urban	Bom Despacho	Central
1149	17-Jun-21	dry	2021	neg	*Callithrix penicillata*	Urban	Belo Horizonte	Metropolitan
1175	17-Jun-21	dry	2021	neg	*Callithrix penicillata*	Urban	Belo Horizonte	Metropolitan
1151	23-Jun-21	dry	2021	neg	*Callithrix penicillata*	Urban	Belo Horizonte	Metropolitan
1153	23-Jun-21	dry	2021	neg	*Callithrix penicillata*	Urban	Belo Horizonte	Metropolitan
1147	25-Jun-21	dry	2021	neg	*Callithrix penicillata*	Urban	Belo Horizonte	Metropolitan
1159	01-Jul-21	dry	2021	neg	*Callithrix penicillata*	Urban	Belo Horizonte	Metropolitan

1167	01-Jul-21	dry	2021	neg	*Callithrix geoffroyi*	Urban	Ipatinga	Rio Doce Valley
1157	05-Jul-21	dry	2021	neg	*Callithrix penicillata*	Urban	Belo Horizonte	Metropolitan
1173	06-Jul-21	dry	2021	neg	*Callithrix penicillata*	Urban	Ribeirão das Neves	Metropolitan
1163	10-Jul-21	dry	2021	neg	*Callithrix penicillata*	Urban	Belo Horizonte	Metropolitan
1181	12-Jul-21	dry	2021	neg	*Callithrix penicillata*	Urban	Belo Horizonte	Metropolitan
1189	14-Jul-21	dry	2021	neg	*Callithrix penicillata*	Urban	Belo Horizonte	Metropolitan
1177	15-Jul-21	dry	2021	neg	*Callithrix penicillata*	Rural	Curvelo	Central
1187	15-Jul-21	dry	2021	neg	*Callithrix penicillata*	Urban	Curvelo	Central
1191	15-Jul-21	dry	2021	neg	*Callithrix penicillata*	Urban	Curvelo	Central
1201	15-Jul-21	dry	2021	neg	*Callithrix penicillata*	Rural	Curvelo	Central
1197	23-Jul-21	dry	2021	neg	*Callithrix penicillata*	Rural	Ubaí	North
1205	04-Aug-21	dry	2021	pos	*Callithrix penicillata*	Urban	Betim	Metropolitan
1195	11-Aug-21	dry	2021	neg	*Callithrix penicillata*	Urban	Belo Horizonte	Metropolitan
1179	16-Aug-21	dry	2021	neg	*Callithrix penicillata*	Peri-urban	Nova Lima	Metropolitan
1199	16-Aug-21	dry	2021	neg	*Callithrix penicillata*	Peri-urban	Nova Lima	Metropolitan
1207	16-Aug-21	dry	2021	neg	*Callithrix penicillata*	Peri-urban	Nova Lima	Metropolitan
1209	16-Aug-21	dry	2021	neg	*Callithrix penicillata*	Peri-urban	Nova Lima	Metropolitan
1185	20-Aug-21	dry	2021	neg	*Callithrix penicillata*	Rural	Sacramento	Triangulo Mineiro
1193	23-Aug-21	dry	2021	neg	*Callithrix penicillata*	Urban	Belo Horizonte	Metropolitan
1183	27-Aug-21	dry	2021	neg	*Callithrix penicillata*	Urban	Santa Luzia	Metropolitan
1203	01-Sep-21	dry	2021	neg	*Callithrix penicillata*	Urban	Paraopeba	Metropolitan
1217	09-Sep-21	dry	2021	neg	*Callithrix penicillata*	Rural	Itaúna	West
1215	15-Sep-21	dry	2021	neg	*Callithrix penicillata*	Urban	Belo Horizonte	Metropolitan
1213	23-Sep-21	dry	2021	neg	*Callithrix geoffroyi*	NA	Santana do Paraíso	Rio Doce Valley
1211	13-Oct-21	rainy	2021	neg	*Cebidae*	Rural	Matozinhos	Metropolitan
1227	21-Oct-21	rainy	2021	neg	*Callithrix penicillata*	Urban	Unaí	Northwest
1224	28-Oct-21	rainy	2021	neg	*Callithrix penicillata*	Urban	Juiz de Fora	Zona da Mata
1225	28-Oct-21	rainy	2021	neg	*Callithrix penicillata*	Urban	Juiz de Fora	Zona da Mata
1219	29-Oct-21	rainy	2021	neg	*Callithrix penicillata*	Urban	Belo Horizonte	Metropolitan
1230	21-Jan-22	rainy	2022	neg	*Callithrix penicillata*	Urban	Sarzedo	Metropolitan

1229	27-Jan-22	rainy	2022	neg	*Callithrix geoffroyi*	Urban	Coronel Fabriciano	Rio Doce Valley
1231	18-Feb-22	rainy	2022	neg	*Callithrix penicillata*	Urban	Ponte Nova	Zona da Mata
1233	03-Mar-22	rainy	2022	neg	*Callithrix penicillata*	Urban	Belo Horizonte	Metropolitan
1232	04-Mar-22	rainy	2022	neg	*Callithrix penicillata*	Urban	Pará de Minas	Metropolitan
1228	08-Mar-22	rainy	2022	pos	*Callithrix penicillata*	Urban	Belo Horizonte	Metropolitan
1235	21-Mar-22	rainy	2022	neg	*Callithrix penicillata*	Urban	Belo Horizonte	Metropolitan
1237	21-Mar-22	rainy	2022	neg	*Callithrix penicillata*	Urban	Papagaios	Metropolitan
1236	23-Mar-22	rainy	2022	neg	*Callithrix penicillata*	Urban	Belo Horizonte	Metropolitan
1239	23-Mar-22	rainy	2022	neg	*Callithrix penicillata*	Urban	Santa Luzia	Metropolitan
1238	20-Apr-22	dry	2022	neg	*Callithrix penicillata*	Urban	Itapajipe	Triangulo Mineiro
1240	27-Apr-22	dry	2022	neg	*Callithrix penicillata*	Urban	Curvelo	Central
1242	27-Apr-22	dry	2022	neg	*Callithrix penicillata*	Urban	Jaboticatubas	Metropolitan
1243	27-Apr-22	dry	2022	neg	*Callithrix penicillata*	Urban	Jaboticatubas	Metropolitan
1245	03-May-22	dry	2022	neg	*Callithrix penicillata*	Urban	Coronel Fabriciano	Rio Doce Valley
1246	03-May-22	dry	2022	neg	*Callithrix geoffroyi*	Urban	Coronel Fabriciano	Rio Doce Valley
1247	03-May-22	dry	2022	neg	*Callithrix penicillata*	Urban	Coronel Fabriciano	Rio Doce Valley
1244	23-May-22	dry	2022	neg	*Callithrix penicillata*	Urban	Contagem	Metropolitan
1248	25-May-22	dry	2022	neg	*Callithrix flaviceps*	Urban	Raul Soares	Zona da Mata
1241	31-May-22	dry	2022	neg	*Callithrix geoffroyi*	Urban	Santana do Paraíso	Rio Doce Valley
1250	31-May-22	dry	2022	neg	*Callithrix penicillata*	Urban	Itabirito	Metropolitan
1252	14-Jun-22	dry	2022	neg	*Callithrix penicillata*	Rural	Augusto de Lima	Central
1249	17-Jun-22	dry	2022	neg	*Callithrix penicillata*	Peri-urban	Augusto de Lima	Central
1251	17-Jun-22	dry	2022	neg	*Callithrix penicillata*	Peri-urban	Augusto de Lima	Central
1253	20-Jun-22	dry	2022	neg	*Callithrix penicillata*	Peri-urban	Augusto de Lima	Central
1268	20-Jun-22	dry	2022	neg	*Callithrix penicillata*	Peri-urban	Augusto de Lima	Central
1271	20-Jun-22	dry	2022	neg	*Callithrix penicillata*	Urban	Belo Horizonte	Metropolitan
1266	21-Jun-22	dry	2022	neg	*Callithrix penicillata*	Peri-urban	Divinópolis	West
1269	21-Jun-22	dry	2022	neg	*Callithrix penicillata*	Peri-urban	Divinópolis	West
1263	22-Jun-22	dry	2022	neg	*Callithrix penicillata*	Urban	Belo Horizonte	Metropolitan
1265	23-Jun-22	dry	2022	neg	*Sapajus spp./Cebidae*	Rural	Raul Soares	Zona da Mata

1261	24-Jun-22	dry	2022	neg	*Callithrix penicillata*	Urban	Contagem	Metropolitan
1273	27-Jun-22	dry	2022	neg	*Callithrix penicillata*	Urban	Ubaí	North
1257	28-Jun-22	dry	2022	neg	*Callithrix geoffroyi*	Urban	Ipatinga	Rio Doce Valley
1270	06-Jul-22	dry	2022	neg	*Callithrix penicillata*	Urban	Betim	Metropolitan
1256	07-Jul-22	dry	2022	neg	*Alouatta caraya*	Urban	Unaí	Northwest
1255	08-Jul-22	dry	2022	neg	*Callithrix geoffroyi*	Urban	Joanésia	Rio Doce Valley
1254	11-Jul-22	dry	2022	neg	*Callithrix penicillata*	Urban	Sete Lagoas	Metropolitan
1264	11-Jul-22	dry	2022	neg	*Callithrix penicillata*	Urban	Sete Lagoas	Metropolitan
1262	12-Jul-22	dry	2022	neg	*Callithrix penicillata*	Urban	Betim	Metropolitan
1267	13-Jul-22	dry	2022	neg	*Callithrix penicillata*	Urban	Augusto de Lima	Central
1259	19-Jul-22	dry	2022	neg	*Callithrix penicillata*	NA	Diamantina	Jequitinhonha
1260	20-Jul-22	dry	2022	neg	*Alouatta guariba clamitans*	rural	Santa Rita de Caldas	South/Southwest
1258	21-Jul-22	dry	2022	neg	*Callithrix penicillata*	Urban	Belo Horizonte	Metropolitan
1281	08-Aug-22	dry	2022	neg	*Callithrix penicillata*	Urban	Belo Horizonte	Metropolitan
1279	12-Aug-22	dry	2022	neg	*Callithrix penicillata*	NA	Itapajipe	Triangulo Mineiro
1275	17-Aug-22	dry	2022	neg	*Callithrix penicillata*	NA	Campo Belo	West
1274	18-Aug-22	dry	2022	pos	*Callithrix penicillata*	Urban	Belo Horizonte	Metropolitan
1278	18-Aug-22	dry	2022	pos	*Callithrix penicillata*	Urban	Belo Horizonte	Metropolitan
1277	05-Sep-22	dry	2022	pos	*Callithrix penicillata*	Urban	Belo Horizonte	Metropolitan
1283	06-Sep-22	dry	2022	pos	*Callithrix penicillata*	rural	Felixlândia	Central
1276	08-Sep-22	dry	2022	pos	*Callithrix penicillata*	Urban	Igarapé	Metropolitan
1286	23-Sep-22	dry	2022	neg	*Sapajus spp./Cebidae*	NA	Caratinga	Rio Doce Valley
1294	28-Sep-22	dry	2022	neg	*Callithrix penicillata*	Urban	Brumadinho	Metropolitan
1295	28-Sep-22	dry	2022	neg	*Callithrix penicillata*	Urban	Brumadinho	Metropolitan
1292	30-Sep-22	dry	2022	neg	*Callithrix geoffroyi*	rural	Santana do Paraíso	Rio Doce Valley
1293	04-Oct-22	rainy	2022	neg	*Callithrix geoffroyi*	Rural	São Pedro Do Suaçuí	Rio Doce Valley
1296	05-Oct-22	rainy	2022	neg	*Callithrix penicillata*	Urban	Belo Horizonte	Metropolitan
1284	06-Oct-22	rainy	2022	neg	*Callithrix penicillata*	Rural	Iraí de Minas	Triangulo Mineiro

1287	26-Oct-22	rainy	2022	neg	*Callithrix penicillata*	Urban	Nova Lima	Metropolitan
1289	23-Nov-22	rainy	2022	neg	*Callithrix penicillata*	Urban	Lagoa Santa	Metropolitan
1285	24-Nov-22	rainy	2022	neg	*Callithrix penicillata*	rural	São Francisco	North
1290	24-Nov-22	rainy	2022	neg	*Callithrix penicillata*	rural	São Francisco	North
1288	13-Dec-22	rainy	2022	neg	*Sapajus spp./Cebidae*	rural	Sacramento	Triangulo Mineiro
1297	10-Jan-23	rainy	2023	neg	*Alouatta fusca clamitans*	NA	Angelândia	Jequitinhonha
1300	16-Jan-23	rainy	2023	neg	*Callithrix penicillata*	NA	Ribeirão Das Neves	Metropolitan
1301	17-Jan-23	rainy	2023	neg	*Callithrix penicillata*	NA	Belo Horizonte	Metropolitan
1298	20-Jan-23	rainy	2023	neg	*Callithrix penicillata*	NA	Belo Horizonte	Metropolitan
1299	31-Jan-23	rainy	2023	neg	*Callithrix penicillata*	NA	Itapajipe	Triangulo Mineiro
1307	31-Jan-23	rainy	2023	neg	*Callithrix penicillata*	NA	Belo Horizonte	Metropolitan
1308	31-Jan-23	rainy	2023	neg	*Callithrix penicillata*	NA	Itapajipe	Triangulo Mineiro
1313	31-Jan-23	rainy	2023	neg	*Callithrix penicillata*	NA	Caxambu	South/Southwest
1302	10-Feb-23	rainy	2023	neg	*Callithrix penicillata*	NA	Nova Lima	Metropolitan
1309	27-Feb-23	rainy	2023	neg	*Callithrix penicillata*	NA	Contagem	Metropolitan
1303	07-Mar-23	rainy	2023	neg	*Callithrix penicillata*	NA	Contagem	Metropolitan
1316	03-Apr-23	dry	2023	neg	*Callithrix penicillata*	NA	Itaúna	West
1318	20-Apr-23	dry	2023	neg	*Callithrix geoffroyi*	NA	Coronel Fabriciano	Rio Doce Valley
1324	27-Apr-23	dry	2023	neg	*Sapajus spp./Cebidae*	NA	Marliéria	Rio Doce Valley
1328	27-Apr-23	dry	2023	neg	*Sapajus spp./Cebidae*	NA	Marliéria	Rio Doce Valley
1331	03-May-23	dry	2023	neg	*Callithrix penicillata*	NA	Rio Acima	Metropolitan
1330	04-May-23	dry	2023	neg	*Callithrix geoffroyi*	NA	Mantena	Rio Doce Valley
1322	17-May-23	dry	2023	neg	*Callithrix geoffroyi*	NA	Mantena	Rio Doce Valley
1329	30-May-23	dry	2023	neg	*Callithrix penicillata*	NA	Belo Horizonte	Metropolitan
1319	21-Jun-23	dry	2023	neg	*Callithrix geoffroyi*	NA	Marliéria	Rio Doce Valley
1325	23-Jun-23	dry	2023	neg	*Callithrix penicillata*	NA	Belo Horizonte	Metropolitan
1326	23-Jun-23	dry	2023	neg	*Callithrix penicillata*	NA	Barbacena	Campo das Vertentes
1327	23-Jun-23	dry	2023	neg	*Callithrix penicillata*	NA	Ibirité	Metropolitan
1320	27-Jun-23	dry	2023	neg	*Callithrix penicillata*	NA	Marliéria	Rio Doce Valley
1321	27-Jun-23	dry	2023	neg	*Callithrix geoffroyi*	NA	Belo Horizonte	Metropolitan

1336	09-Aug-23	dry	2023	neg	*Callithrix penicillata*	NA	Belo Horizonte	Metropolitan
1334	14-Aug-23	dry	2023	neg	*Callithrix penicillata*	NA	Ibirité	Metropolitan
1333	16-Aug-23	dry	2023	neg	*Callithrix geoffroyi*	NA	Novo Cruzeiro	Jequitinhonha
1332	17-Aug-23	dry	2023	neg	*Callithrix penicillata*	NA	Jaboticatubas	Metropolitan
1335	17-Aug-23	dry	2023	neg	*Callithrix penicillata*	NA	Jaboticatubas	Metropolitan
1161	NA	NA	2021	neg	*NA*	NA	NA	NA
1221	NA	NA	2021	neg	*NA*	NA	NA	NA
1223	NA	NA	2021	neg	*NA*	NA	NA	NA
1234	NA	NA	2022	neg	*NA*	NA	NA	NA
1272	NA	NA	2022	neg	*NA*	NA	NA	NA
1280	NA	NA	2022	pos	*NA*	NA	NA	NA
1282	NA	NA	2022	pos	*NA*	NA	NA	NA
1291	NA	NA	2022	neg	*NA*	NA	NA	NA
1304	NA	NA	2023	neg	*Callithrix spp.*	NA	Ibirité	Metropolitan
1305	NA	NA	2023	neg	*Callithrix spp.*	NA	Belo Horizonte	Metropolitan
1306	NA	NA	2023	neg	*Callithrix spp.*	NA	Belo Horizonte	Metropolitan
1310	NA	NA	2023	neg	*Callithrix spp.*	NA	Belo Horizonte	Metropolitan
1311	NA	NA	2023	neg	*Callithrix spp.*	NA	Belo Horizonte	Metropolitan
1312	NA	NA	2023	neg	*Callithrix spp.*	NA	Matias Barbosa	Zona da Mata
1314	NA	NA	2023	neg	*Callithrix spp.*	NA	Confins	Metropolitan
1315	NA	NA	2023	neg	*Callithrix spp.*	NA	Belo Horizonte	Metropolitan
1317	NA	NA	2023	neg	*Callithrix spp.*	NA	Santana Do Paraíso	Rio Doce Valley
1323	NA	NA	2023	neg	*Callithrix spp.*	NA	Rio Acima	Metropolitan

Neg: negative. Pos: positive. NA: not available.
